# Plant–fungus competition for nitrogen erases mycorrhizal growth benefits of *Andropogon gerardii* under limited nitrogen supply

**DOI:** 10.1002/ece3.2207

**Published:** 2016-05-30

**Authors:** David Püschel, Martina Janoušková, Martina Hujslová, Renata Slavíková, Hana Gryndlerová, Jan Jansa

**Affiliations:** ^1^ Laboratory of Fungal BiologyInstitute of MicrobiologyCzech Academy of SciencesPragueCzech Republic; ^2^ Department of Mycorrhizal SymbiosesInstitute of BotanyCzech Academy of SciencesPrůhoniceCzech Republic

**Keywords:** Arbuscular mycorrhizal fungi, belowground carbon drain, inoculation, mycorrhizal benefits and costs, nutrient uptake response, shoot nitrogen‐to‐phosphorus ratio

## Abstract

Considered to play an important role in plant mineral nutrition, arbuscular mycorrhizal (AM) symbiosis is a common relationship between the roots of a great majority of plant species and glomeromycotan fungi. Its effects on the plant host are highly context dependent, with the greatest benefits often observed in phosphorus (P)‐limited environments. Mycorrhizal contribution to plant nitrogen (N) nutrition is probably less important under most conditions. Moreover, inasmuch as both plant and fungi require substantial quantities of N for their growth, competition for N could potentially reduce net mycorrhizal benefits to the plant under conditions of limited N supply. Further compounded by increased belowground carbon (C) drain, the mycorrhizal costs could outweigh the benefits under severe N limitation. Using a field AM fungal community or a laboratory culture of *Rhizophagus irregularis* as mycorrhizal inoculants, we tested the contribution of mycorrhizal symbiosis to the growth, C allocation, and mineral nutrition of *Andropogon gerardii* growing in a nutrient‐poor substrate under variable N and P supplies. The plants unambiguously competed with the fungi for N when its supply was low, resulting in no or negative mycorrhizal growth and N‐uptake responses under such conditions. The field AM fungal communities manifested their potential to improve plant P nutrition only upon N fertilization, whereas the *R*. *irregularis* slightly yet significantly increased P uptake of its plant host (but not the host's growth) even without N supply. Coincident with increasing levels of root colonization by the AM fungal structures, both inoculants invariably increased nutritional and growth benefits to the host with increasing N supply. This, in turn, resulted in relieving plant P deficiency, which was persistent in non‐mycorrhizal plants across the entire range of nutrient supplies.

## Introduction

Arbuscular mycorrhizal (AM) symbiosis is a relationship established between the majority of terrestrial plant species and fungi from the phylum Glomeromycota (Smith and Read 2008). One of the principal benefits the host plants derive from this association is enhanced uptake of soil nutrients. The fine AM fungal hyphae form dense networks in soils, increasing surface area per unit biomass by as much as two orders of magnitude greater than plant roots alone (Raven and Edwards 2001). AM fungi reach well beyond the nutrient depletion zone of the roots and enhance uptake of soil nutrients having limited mobility, such as phosphorus (P) and zinc (Jansa et al. 2003, 2011; Kiers et al. 2011). In addition, uptake of nitrogen (N) from soil to plant via the AM fungal hyphae has been demonstrated (Mäder et al. 2000). The impact of this symbiosis on plant N acquisition probably remains limited, however, to specific conditions wherein N is not very mobile or not directly available to plants yet is abundant in the soil, such as ${\text{NH}}_{4}^{+}$ or organic forms of N (Govindarajulu et al. 2005; Cruz et al. 2007; Barrett et al. 2011; Hodge and Storer 2015). The price that plants pay to be provided the mycorrhizal benefits is to supply the fungi with reduced carbon (C) compounds, most likely in the form of simple sugars (Kiers et al. 2011; Casieri et al. 2013; Konvalinková et al. 2015). Depending upon the absolute amounts of nutrients transferred in one direction and of C in the other, as well as upon the environmental context (soil nutrient availability, light intensity, various kinds of stresses), the growth response of plants to mycorrhiza may vary from positive to negative (Johnson et al. 1997, 2015).

It is important to understand what are the drivers that determine plants’ mycorrhizal growth response (MGR). Among the obvious, well‐known factors are the plant's identity and its demand for various resources that may quantitatively vary from plant to plant (Hoeksema et al. 2010), functional diversity of AM fungi on a species or even isolate level (Klironomos 2003; Munkvold et al. 2004), fungal adaptations to specific site conditions (Malcová et al. 2003), and certain local plant adaptations (Klironomos 2003; Pánková et al. 2011). If a plant's growth is limited by a certain resource and AM symbiosis assists in gathering that resource, then the plant is likely to respond positively to the presence of the AM fungus. This notion is based upon the “law of the minimum”, a concept formulated nearly 200 years ago (van der Ploeg et al. 1999) and predicting that the growth of a plant is limited by the one resource most scarce in relation to the plant's needs. It could also be formulated as “the availability of the most abundant (essential) nutrient in the soil is only as good (i.e., helpful for plant growth) as the availability of the least abundant (essential) nutrient in the soil”. Simply and metaphorically, this might be stated as “a chain is only as strong as its weakest link”.

Mycorrhizas generally and the AM symbiosis in particular have traditionally been viewed reductively and regarded merely as pumps mediating efficient P uptake from soil to plants. Consequently, plants were expected to respond more positively to mycorrhizal symbiosis in soils with limited P availability than in soils with ample P (Smith and Read 2008). Whereas improved P uptake of the host plant is without a doubt the most important ecosystem‐relevant feature of AM symbiosis (van der Heijden et al. 2008), uptake of N to plants via AM hyphae can reach significant levels under some circumstances (Johansen et al. 1992; Hodge et al. 2010; Fellbaum et al. 2012), and particularly under conditions of very high soil availability of P (Blanke et al. 2005).

Consistent with the “law of the minimum” concept, numerous pieces of evidence have appeared in the past decade showing that the sheer quantity of nutrients is not the most important determinant of plant MGR. Instead, the actual stoichiometry of all resources potentially limiting plant growth (individual nutrients, water, light) and whether mycorrhiza could relieve such limitation has turned out to be the keys to understanding plants’ context dependency on mycorrhizal symbiosis (Hoeksema et al. 2010; Johnson et al. 2015). Depending on the relative availability of soil nutrients such as P and N on the one hand and light and CO_2_ (i.e., the primary determinants of availability of reduced C generated through photosynthesis) on the other, the trade of mineral nutrients for C between the partners within the mycorrhizal symbiosis could result in a whole range of apparent effects for the host plants, extending from mutualism to commensalism and to parasitism (Johnson et al. 1997, 2015; Johnson 2010). The greatest mycorrhizal benefits to its host plant in terms of positive MGR have been observed particularly under P limitation, but only when such other resources as N and light were not limiting (Johnson 2010; and references therein). This conditionality of the different resource availability on the outcome (phenotype) of the symbiosis has been explained by the so‐called “trade balance model”, predicting that the functioning of AM symbiosis is determined by the interaction of N and P availabilities with the C supply and demand among plants and fungi (Johnson 2010). This concept has recently been verified experimentally by manipulating nutrient and light availabilities to *Andropogon gerardii* plants grown in three different grassland soils (Johnson et al. 2015). Although previous experiments were strongly suggestive of competition for N between the plants and the AM fungi (Hodge and Fitter 2010; Hodge and Storer 2015; Johnson et al. 2015), unequivocal proof of such competition is still missing in the published literature.

To close this gap, in this study, we addressed several hypotheses related to the trade balance model. We predicted that (1) with increasing relative availability of N to plants mycorrhizal benefits as well as root colonization levels would increase. In this respect, we were particularly curious to determine whether AM fungi would improve plant N acquisition even under N‐limiting conditions or increase uptake by plants only if N became more available. (2) In contrast, we hypothesized that with increasing availability of P both mycorrhizal benefits and root colonization would decrease. (3) Due to tight coupling of mycorrhizal costs and benefits (Kiers et al. 2011; Fellbaum et al. 2014), we predicted higher belowground C drain in conditions where a mycorrhiza is very important for plant functioning (i.e., when plants show higher MGR) compared to conditions of lower importance of mycorrhizal symbiosis (i.e., when the MGR is low or negative). To quantify belowground C drain, we carried out a pulse‐chase labeling experiment using ^13^CO_2_ to feed the plants and quantified the redistribution of ^13^C between shoots, roots, and soil. To test the above hypotheses, we devised a pot experiment using *A. gerardii* as a model host because it is a highly mycorrhiza‐responsive plant and it has been used previously to verify the trade balance model as a framework for explaining context dependency of mycorrhizal functioning (Johnson et al. 2015). The grass was grown in a sterilized substrate with inherent low fertility, with or without the mycorrhiza, and with nutrient inputs manipulated through mineral fertilizer application. We used nonsterile field soil as complex microbial inoculum including mycorrhiza and compared it to inoculation with soil where the indigenous mycorrhiza had been inactivated (Experiment 1). To confirm the findings reached using this complex soil inoculum, we replicated part of the experiment using just a single AM fungal isolate inoculum consisting of *R. irregularis* “Chomutov” (Experiment 2).

## Material and Methods

### Experimental design

In Experiment 1, *A. gerardii* was exposed to a fertilization gradient comprising four levels of nutrient inputs. The plants were inoculated either with a nonsterile field soil containing indigenous AM fungal communities together with other soil microbes or with the same soil devoid of the indigenous AM fungi (blank inoculum). An Experiment 2 accompanied the complete experimental design outlined above to verify the results obtained with complex soil inoculation by means of those obtained with a single AM fungal isolate *R. irregularis* “Chomutov” (or respective blank inoculum) previously grown in a laboratory culture. This was important mainly because of other microbes (e.g., plant pathogens) present in nonsterile soil, which could potentially exert a variety of other than symbiotic effects on the plants and thus conceal the effect of the AM fungi. Experiment 2 was conducted in a reduced design comprising just two fertilization treatments, but otherwise under exactly the same conditions as in Experiment 1. Five replicates per treatment meant there were 60 pots in total (40 and 20 pots for Experiments 1 and 2, respectively), and these were arranged in a fully randomized layout.

### Mycorrhizal inocula

#### Experiment 1

The plants were inoculated with nonsterile soil that had been obtained from a suburban meadow near Litoměřice, Czech Republic (50°31′54.53′′N, 14°06′37.10′′E), <4 weeks prior to establishment of the experiment. The soil was air‐dried and sieved through a 4 mm sieve. The nonsterile soil inoculum contained AM fungal genera *Funneliformis*,* Septoglomus*,* Rhizophagus*,* Claroideoglomus*,* Gigaspora*, and *Diversispora*, as revealed by molecular analysis of root‐borne AM communities in a preliminary glasshouse experiment (Řezáčová et al. 2016).

A blank (non‐mycorrhizal) inoculum was prepared through mild heat disinfection (pasteurization) of previously air‐dried, sieved, and rewetted (50% of field capacity) field soil (3× at 80°C for 12 h each, 24 h apart), resulting in elimination of the indigenous AM fungi and other soil microbes. Microbial communities (other than AM fungi) were reconstituted through inoculation of the heat‐treated soil with 100 mL·kg^−1^ of nonsterile field soil filtrate (soil slurry 1:10, w:v, passed through a 40‐*μ*m sieve) and incubation at laboratory temperature for 2 weeks. Every pot was inoculated with 50 g of either the nonsterile soil or the blank inoculum applied as a layer 6–8 cm below the pot's soil surface.

#### Experiment 2

The plants were inoculated with the AM fungal isolate *R*. *irregularis* “Chomutov” (treatment hereafter referred to as Ri+) or with the respective blank inoculum (Ri−). The AM isolate used here originated from a spoil bank of the Merkur opencast coal mine (Chomutov, North Bohemian Coal Basin, Czech Republic). As this isolate is known from our previous experience (Janoušková et al. 2013) to be highly infective and able to colonize a wide range of host plants, it was considered a highly suitable reference for the mycorrhizal communities contained in the nonsterile soil and used for inoculation of the plants in the Experiment 1. The isolate was maintained at the AM fungal collection of the Department of Mycorrhizal Symbioses (Institute of Botany, Czech Academy of Sciences, Czech Republic) in the same sand–zeolite–soil substrate as used in both experiments. The inoculum cultures, established with *Zea mays* as the initial and *Desmodium* sp. as the follow‐up host plants, were 11 months old when used as inoculum source. Inspection under a binocular magnifier had confirmed very abundant intraradical and extraradical sporulation of *R*. *irregularis*, as well as an absence of contamination by other AM fungal morphospecies. To prepare the Ri+ inoculum, the shoots were removed and the roots were cut into ca 1 cm pieces and mixed back into the substrate, which was then dried at room temperature for 2 weeks.

The Ri− blank inoculum was produced in exactly the same manner, but using blank (non‐mycorrhizal) cultures: the same plants were grown in the same substrate and cultivation conditions, but without AM fungi. Inspection of blank cultures under a binocular magnifier revealed an absence of AM fungal spores and mycelium aggregates. The same dosage and application were used as described for Experiment 1.

### Substrate

The substrate used in the study consisted of autoclaved (at 121°C for 30 min) quartz sand with grain size <4 mm, autoclaved zeolite (Zeopol s.r.o., Břeclav, Czech Republic; www.zeopol.com) with grain size 1–2.5 mm, and *γ*‐irradiated (>25 kGy) soil from Litoměřice, Czech Republic (pH_H2O_ 7.88, 42% clay, 40% sand, total P 797 mg·kg^−1^, water‐extractable P 3.3 mg·kg^−1^, organic C 2.26%, total N 0.13%) thoroughly mixed together in a ratio of 9:9:2 (v:v:v). The soil was the same as that used for the inocula preparations.

The substrate was filled into tall, 2‐L plastic pots (11 × 11 × 20 cm). First, a bottom layer 14 cm deep was filled, then a thin layer of evenly spread soil (inoculum or blank) was applied, and finally a 6‐cm top layer of substrate was added.

### Plant cultivation

**Figure 1:**
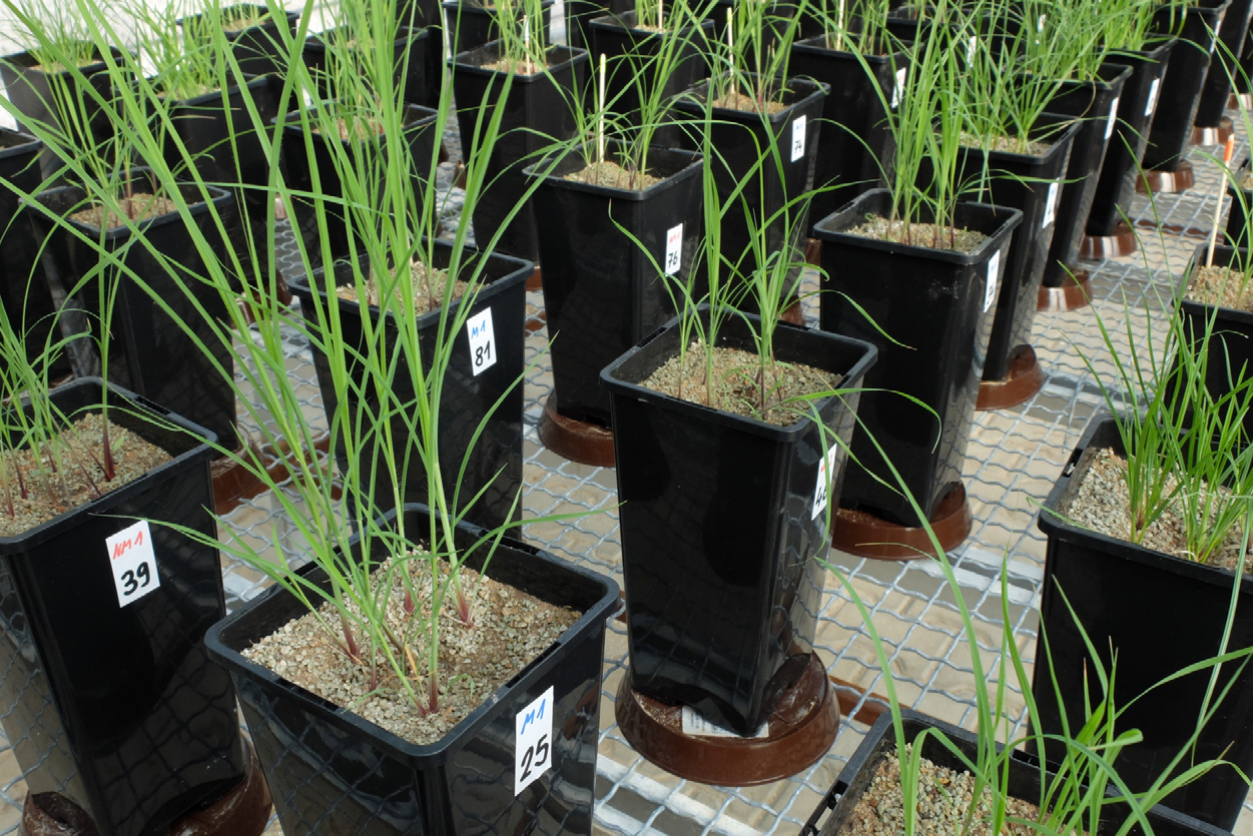
*Andropogon gerardii* Vitman, known commonly as big bluestem, is a C_4_ grass native to North America. It is highly responsive to mycorrhiza formation, especially under low P availability in the soil (Noyd et al. 1995).

The experiments were conducted in a greenhouse without supplementary lighting during high summer, from the end of June to the beginning of September (10 weeks in total). Temperatures ranged from 20 to 35°C during the day and did not drop below 15°C at night. Each pot was sown with 21 seeds of *A. gerardii* (Jelitto Staudensamen GmbH, Schwarmstedt, Germany). During the third week after sowing, plants were thinned to 7 per pot (Fig. 1).

Throughout the experiments, the pots were watered with distilled water (all pots received 25, 50, or 100 mL·day^−1^, depending on plant age and the weather). During hot summer days, additional watering was carried out as necessary.

### Fertilization

From the third week after sowing through the course of the experiments, the plants were fertilized weekly with liquid fertilizer for eight consecutive weeks. Four fertilization treatments were considered in Experiment 1: (1) the nonfertilized treatment (hereafter referred to as N0 P0), where plants received 50 mL of distilled water instead of nutrient input; (2) basic fertilization (N1 P1), providing each pot with a modified “P2N3 solution” (Gryndler et al. 1992), in which the P concentration was further reduced to 20% of the original recipe and plants thus received 6.827 mg of N (in nitrate form) and 0.054 mg of P (in orthophosphate form) per application; (3) a triple‐N treatment (N3 P1), where N input was increased to 20.481 mg per application by adding the corresponding amount of NaNO_3_ to the N1 P1 nutrient solution; and (4) a triple‐N triple‐P treatment (N3 P3), where KH_2_PO_4_ was added to the N3 P1 solution to increase P input to 0.163 mg per application per pot. In every case, 50 mL of the respective nutrient solution was applied per pot each week. The nutrient inputs throughout the experiment roughly matched agricultural application rates of 55 kg (in the N1 treatment) and 160 kg (in the N3 treatment) of N and 0.43 kg (in the P1 treatment) and 1.3 kg (in the P3 treatment) of P per hectare over the duration of the experiment.

Experiment 2 was conducted in a reduced design comprising just two fertilization treatments, N0 P0 and N1 P1 as described above.

### ^13^CO_2_ labeling

To track C flow from the shoots to the roots and substrate, plants were labeled with ^13^CO_2_ 3 days prior to harvest using a hermetic 0.75 m^3^ Plexiglas chamber. Due to the chamber's space limitations and reasons of logistics (harvest planning), labeling was conducted in two runs 2 days apart. Temperature, humidity, and CO_2_ concentration inside the chamber were monitored during labeling (Testo 435‐2; Testo AG, Lenzkirch, Germany). First, plants were left to consume most of the CO_2_ contained in the air inside the chamber. Once the ambient CO_2_ was depleted below 150 *μ*mol·mol^−1^, the air in the chamber was enriched with ^13^CO_2_ by injecting 25 mL of 20% orthophosphoric acid onto 1 g of 99% ^13^C‐enriched calcium carbonate powder (Sigma–Aldrich, Buchs, Switzerland) placed previously in a beaker inside the chamber. The air circulation inside the chamber was maintained by an internal fan. Labeling took 1 h and always was completed at noon. Weather conditions during labeling were partly cloudy on both labeling days.

### Harvest and sampling

The plants were harvested 3 days after ^13^CO_2_ labeling, corresponding to 75 and 78 days after sowing. First, the shoots were cut at the substrate surface level, pooled per pot, dried at 65°C to constant weight, and weighed to obtain shoot dry weight (SDW).

Thereafter, the compact root system with substrate was removed from the pot. Mycorrhizal colonization was assessed using roots collected from a depth of 4–8 cm. This horizon was cut from the rest of the root system and further processed separately: roots were thoroughly washed of substrate, cut into ca 1 cm pieces, and homogenized in a beaker with 1 L of water. Then, a subsample of homogenized roots was taken, weighed fresh, and placed into 50% ethanol. The remaining roots were also weighed fresh and then reweighed after drying at 65°C to constant weight. The remaining top and bottom parts of the root system were also washed of substrate and weighed fresh. Overall root dry weight (RDW) of the entire root system per pot was then calculated. Plants’ total dry weight (TDW) was then calculated as the sum of SDW and RDW.

To compare plants’ growth response to inoculation in different treatments, MGR was calculated for TDW according to the equation MGR = (M − NM_mean_)/NM_mean_ × 100% (Gange and Ayres 1999), where M is the TDW recorded for a given inoculated pot and NM_mean_ is the mean TDW of pots in the corresponding noninoculated treatment.

Root samples stored temporarily in ethanol were stained using the modified method of Koske and Gemma (1989): the roots were first macerated in 10% KOH (60 min at 90°C, followed by 25 min at room temperature), then washed with tap water, neutralized in 2% lactic acid (30 min at 90°C), and stained with 0.05% Trypan blue in LG (lactic acid–glycerol–water, 1:1:1, v:v:v) for 30 min at 90°C plus overnight at room temperature. The next day, the roots were washed with tap water and further stored in LG. Colonization was quantified under a dissecting microscope at 100× magnification following the method of McGonigle et al. (1990). One hundred root intersections with the eyepiece grid were observed per root sample while recording separately the occurrence of AM fungal hyphae, arbuscules (highly branched haustoria‐like structures formed by the AM fungi inside root cortical cells where a significant part of the symbiotic exchange of nutrients for C is taking place (Rausch et al. 2001)), and vesicles (spore‐like swellings of the AM hyphae inside the roots, serving for fungal storage and survival (Kiers et al. 2011)).

### Elemental analyses

Prior to analyses of P, N, and C in plant tissues and substrate, the samples of shoot or root dry matter or substrate were ground to powder using a ball mill (MM200; Retsch, Haan, Germany). Phosphorus concentrations in the plant biomass were analyzed using the Malachite Green method (Ohno and Zibilske 1991) after wet digestion of the samples in concentrated HNO_3_ at 150°C for 1.75 h using a Tecator Digestion System 40 (Foss, Hillerød, Denmark).

The N and C concentrations and isotopic composition of these elements in shoots, roots, and substrate (the last for C only) were measured using a Flash EA 2000 elemental analyzer coupled with a Delta V Advantage isotope ratio mass spectrometer (Thermo Fisher Scientific, Waltham, MA). Belowground drain of ^13^C (^13^C BGD) was calculated as (^13^C_R_ + ^13^C_T_)/^13^C_S_, where ^13^C_R__(T and S)_ represented ^13^C excess (mmol), that is, the amount of ^13^C originating from the ^13^C pulse labeling, calculated as in Konvalinková et al. (2015), in roots, substrate, and shoots, respectively.

Total N and P contents were calculated from SDW and RDW and concentrations of the given element in shoot and root biomass, respectively. Additionally, “mycorrhizal N‐uptake response” (MNR) and “mycorrhizal P‐uptake response” (MPR) were calculated similarly as MGR. Shoot N:P ratio was calculated as an indicator of plant nutrient limitation, considering N:P < 14 to indicate plant N limitation and N:P > 16 to indicate plant P limitation (Koerselman and Meuleman 1996).

### Statistical analyses

The data were analyzed using STATISTICA 12 (StatSoft Inc., Tulsa, OK) and StatPlus 2009 (AnalystSoft Inc., Walnut, CA). All presented data passed tests for normality and homogeneity of variance (if not stated otherwise). The data on mycorrhizal colonization were analyzed with one‐way ANOVA followed by Tukey's HSD test for separation of means.

The data for SDW, RDW, TDW, root‐to‐shoot biomass (R:S) ratio, ^13^C BGD, shoot N:P ratio, and N and P contents and concentrations in plant tissues were first analyzed with two‐way ANOVA to determine the contribution of individual factors (fertilization and inoculation) to explaining the variability. Subsequently, a *t*‐test was used to test the differences between inoculation pairs for each fertilization treatment. The data on shoot and root P concentrations and total P content showed low homogeneity of variances, which was improved by neither square root nor logarithmic transformation. It appears that in this particular case, the low homogeneity of data variance across all treatments was caused by considerable differences between experimental treatments (i.e., strong effect of inoculation). Because the data were distributed normally and also for the sake of consistency with other statistical analyses, we subjected all P‐related data to standard parametric analyses rather than to use nonparametric tests.

The data on MGR, MNR, and MPR were analyzed using a *t*‐test for single means against zero (which represented the mean of the respective noninoculated treatment). Significant differences between means were then identified based on multiple comparisons according to Tukey's HSD test. The correlation analyses between selected plant parameters were carried out across both experiments using a linear regression model.

## Results

### Mycorrhizal colonization

**Figure 2:**
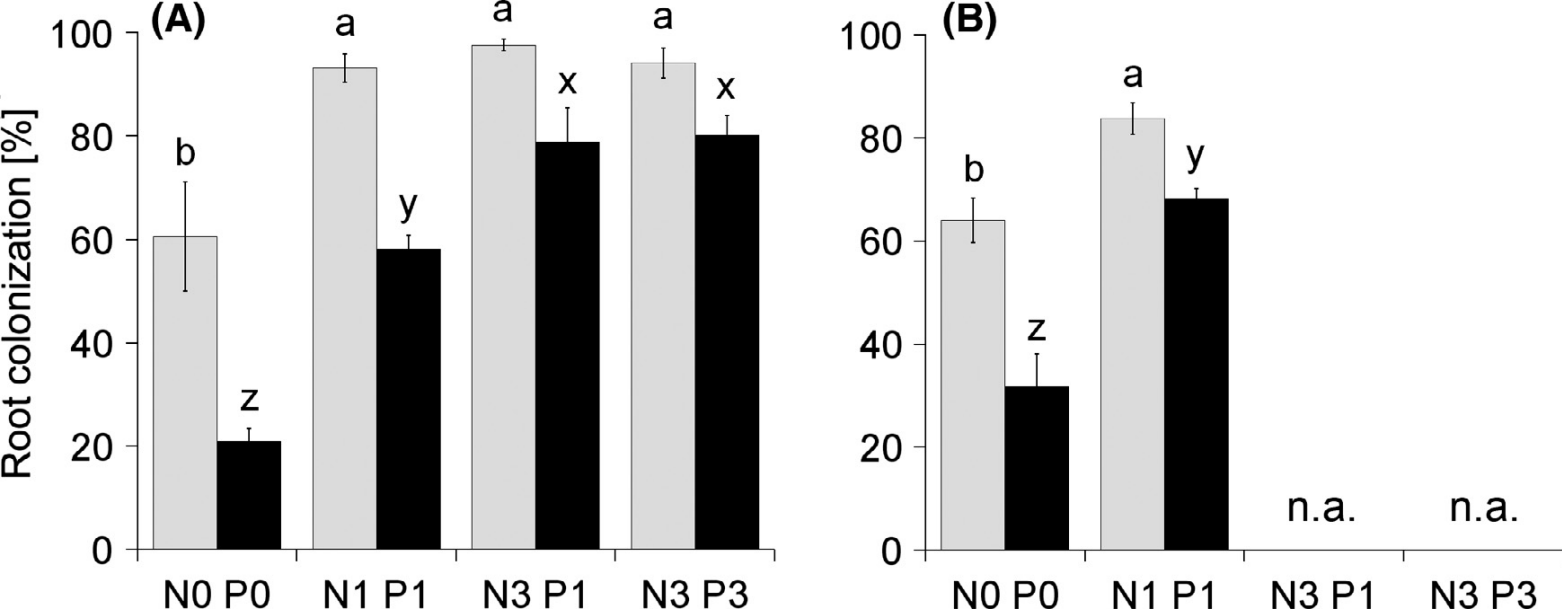
Mycorrhizal colonization of *Andropogon gerardii* roots inoculated with native soil and grown under four fertilization treatments – Experiment 1 (A), or inoculated with *Rhizophagus irregularis* and grown under two fertilization treatments – Experiment 2 (B). Each bar represents the mean (*N* = 5, ±SE) of the percentage of root length colonized by mycorrhizal hyphae (gray columns) and arbuscules (black columns) as per the gridline intersection method (McGonigle et al. 1990). Treatment labels on the horizontal axis consist of the element concerned (nitrogen, N; phosphorus, P) and its input dose (zero, single, or triple); n.a. – not applicable, treatment not included in this experiment. Different letters above columns indicate significant differences according to Tukey's HSD test (*P *<* *0.05) following significant ANOVA, separately for each of the two variables.

All plants inoculated either with nonsterile soil or with the *R*. *irregularis* isolate had their roots highly colonized (Fig. 2A and B, respectively), while zero or negligible levels of mycorrhizal colonization (<1% root length colonized) were observed in the roots of non‐mycorrhizal plants. Fertilization had a highly significant (*P *<* *0.001) positive effect on both hyphal colonization and the incidence of arbuscules in the roots. Mycorrhizal colonization thus increased consistently along the N fertilization gradient, with only a limited influence of the P fertilization (contrast between the N3 P1 and N3 P3 treatments in Experiment 1 was found to be not significant, Fig. 2A). In particular, the incidence of arbuscules in the roots increased fourfold with increasing N inputs. No significant differences due to fertilizer inputs were found for the incidence of vesicles, which ranged from ca 20% to 40% of root length across all treatments (data not shown).

### Plant growth and C allocation

#### Experiment 1

Results of two‐way ANOVA evaluating the effects of experimental factors on the measured growth parameters and allocation of recently fixed C between shoots and roots of *A. gerardii* showed a similar pattern for most parameters: highly significant effects of fertilization and inoculation as well as their interaction (Table 1). A single significant effect of inoculation was recorded for the RDW.

**Table 1:** Effects of the two experimental factors (Fertilization, four levels, and mycorrhizal Inoculation, two levels) and their interaction on various parameters of *Andropogon gerardii* plants grown in Experiment 1 as per the two‐way analysis of variance. *F*‐values and statistical significance levels are shown

Parameter	Factor	*F*	*P*
Total dry weight	(1) Fertilization	3.08	*
	(2) Inoculation	43.12	***
	(1) × (2)	9.26	***
Shoot dry weight	(1) Fertilization	10.96	***
	(2) Inoculation	96.96	***
	(1) × (2)	25.46	***
Root dry weight	(1) Fertilization	0.59	ns
	(2) Inoculation	15.96	***
	(1) × (2)	2.44	ns
R:S dry biomass ratio	(1) Fertilization	11.59	***
	(2) Inoculation	16.92	***
	(1) × (2)	18.55	***
Belowground ^13^C drain	(1) Fertilization	7.76	***
	(2) Inoculation	5.93	*
	(1) × (2)	12.26	***
Shoot N:P ratio	(1) Fertilization	29.45	***
	(2) Inoculation	234.15	***
	(1) × (2)	8.97	***
Total N content	(1) Fertilization	89.58	***
	(2) Inoculation	101.29	***
	(1) × (2)	37.74	***
Shoot N concentration	(1) Fertilization	132.51	***
	(2) Inoculation	13.70	***
	(1) × (2)	6.84	**
Root N concentration	(1) Fertilization	84.94	***
	(2) Inoculation	0.55	ns
	(1) × (2)	4.10	*
Total P content	(1) Fertilization	12.85	***
	(2) Inoculation	132.69	***
	(1) × (2)	18.14	***
Shoot P concentration	(1) Fertilization	11.04	***
	(2) Inoculation	106.86	***
	(1) × (2)	8.79	***
Root P concentration	(1) Fertilization	5.04	**
	(2) Inoculation	141.47	***
	(1) × (2)	11.17	***

*0.01 ≤ *P *<* *0.05; **0.001 ≤ *P *<* *0.01; ***P <* *0.001; ns, *P *≥* *0.05.

**Figure 3:**
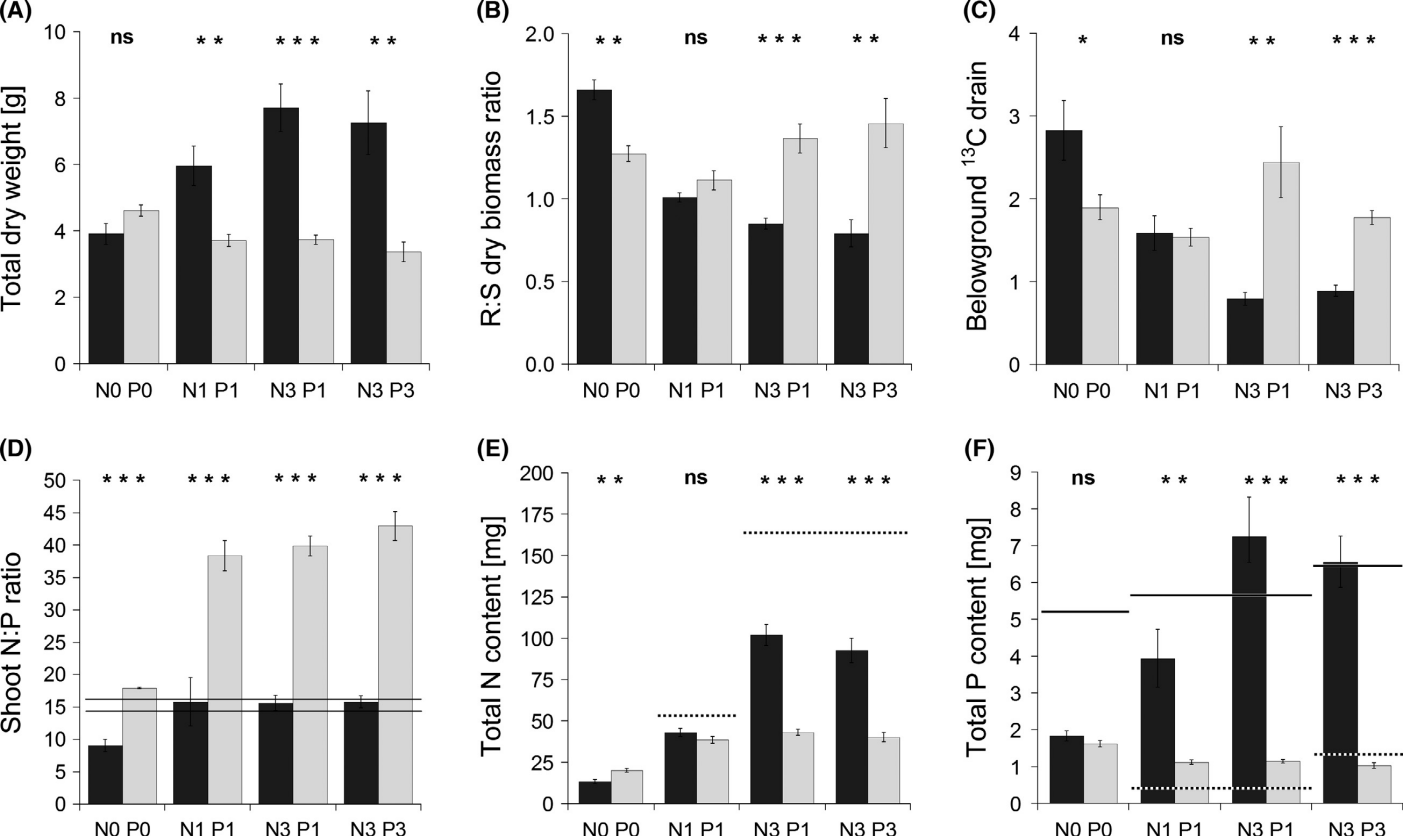
Total (root + shoot) dry biomass (A), root‐to‐shoot (R:S) dry biomass ratio (B), belowground ^13^C drain (C), shoot nitrogen‐to‐phosphorus content ratio (D), total nitrogen (N) content (E), and total phosphorus (P) content (F) of *Andropogon gerardii* plants grown in Experiment 1 under four fertilization treatments, either with a native soil mycorrhizal community (dark columns) or with a respective blank inoculum (light columns). Treatment labels on the horizontal axis consist of the element (N or P) concerned and its input dose (zero, single, or triple). Each bar represents the mean of five replicates (±SE). Symbols above the columns indicate the significance of the contrast between the mycorrhizal and non‐mycorrhizal plants for each fertilization treatment according to the *t*‐test (*0.01 ≤ *P *<* *0.05; **0.001 ≤ *P *<* *0.01; ***P <* *0.001; ns, *P *≥* *0.05). The lower horizontal line in graph D indicates the level of shoot N:P ratio below which the plants are considered N‐limited, whereas the upper line indicates the N:P ratio above which they are considered P‐limited according to Koerselman and Meuleman (1996). Between these lines, the plants are considered to be colimited by both nutrients. The dotted horizontal lines in graphs E and F indicate the levels of N and P inputs, respectively, from the fertilizers in each of the fertilization treatments. The solid lines in graph F indicate the levels of available (water‐extractable) P content in the experimental pots.

**Figure 4:**
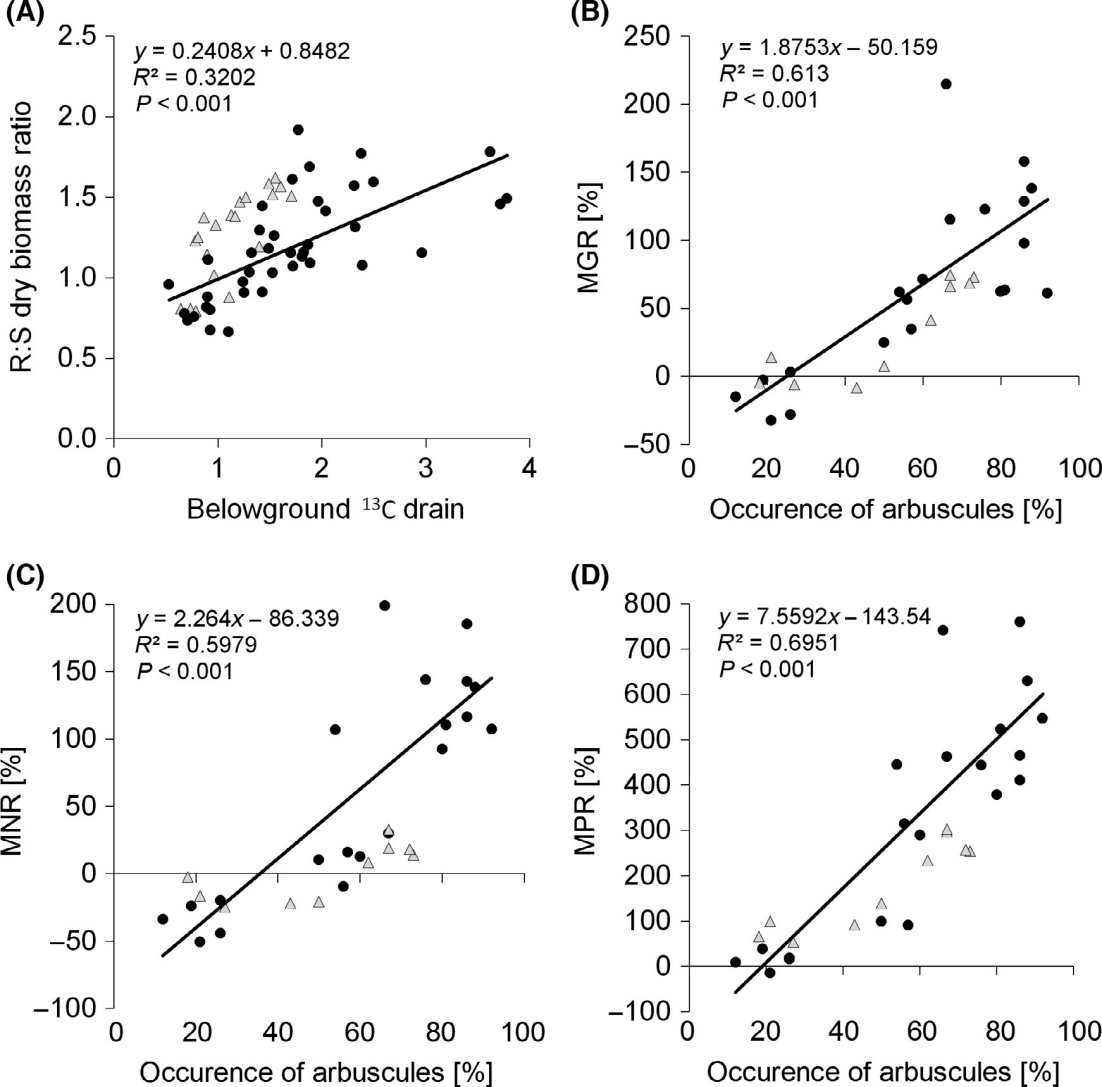
Correlation between the root‐to‐shoot (R:S) dry biomass ratio and belowground ^13^C drain (A) and correlations between the fractional root length colonized by arbuscules and either the mycorrhizal growth response – MGR (B), mycorrhizal nitrogen (N)‐uptake response – MNR (C) or mycorrhizal phosphorus (P)‐uptake response – MPR (D) of *Andropogon gerardii* plants. The circles and triangles represent Experiments 1 and 2, respectively. The correlations were calculated using the data from both experiments together. Statistical significance (*P*‐) values were derived from a goodness‐of‐fit test of the linear regression model.

**Figure 5:**
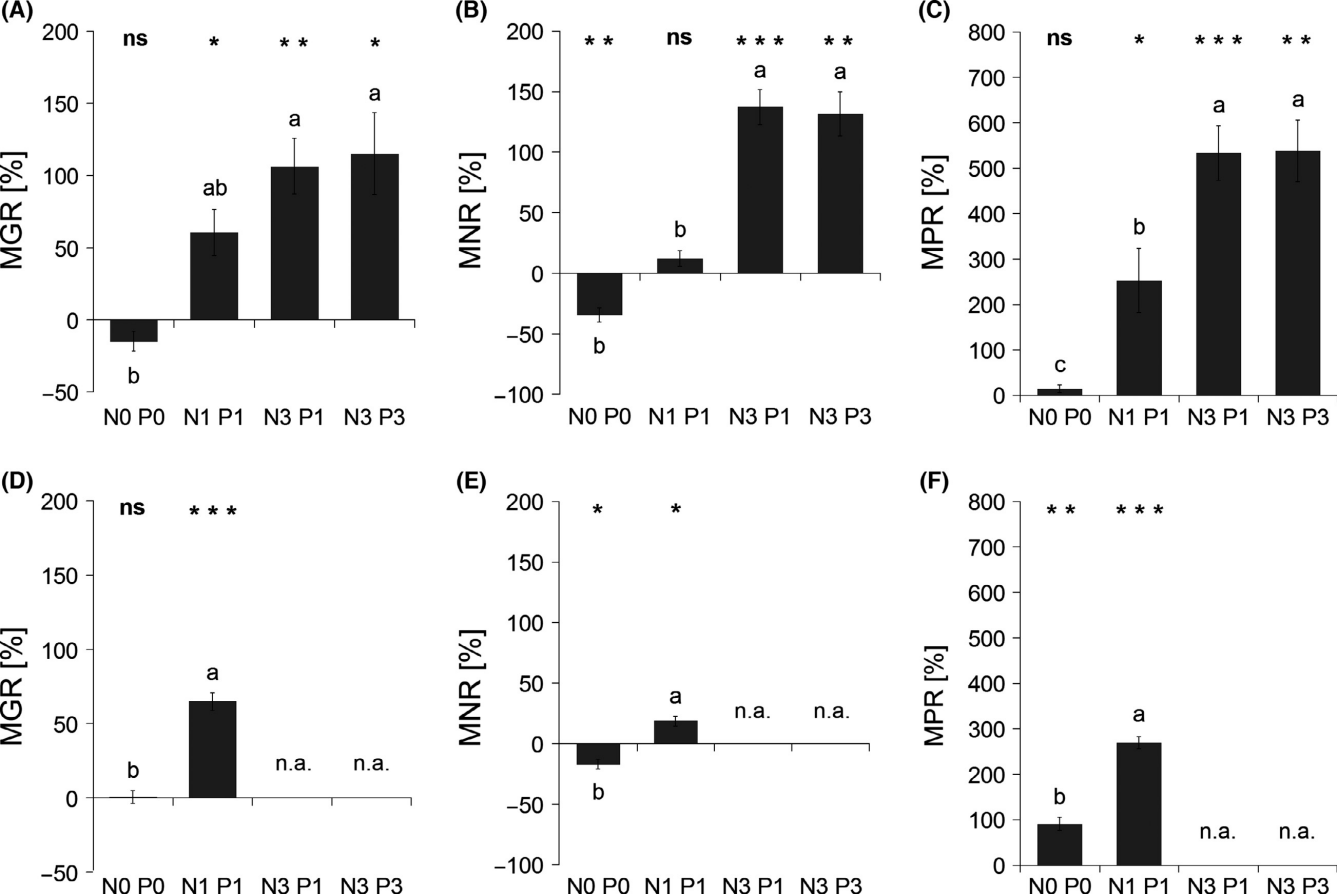
Mycorrhizal growth response – MGR, mycorrhizal nitrogen (N)‐uptake response – MNR, and mycorrhizal phosphorus (P)‐uptake response – MPR of *Andropogon gerardii* either to the presence of native mycorrhizal communities under four fertilization treatments – Experiment 1 (A–C), or to the presence of *Rhizophagus irregularis* under two fertilization treatments – Experiment 2 (D–F). Response is expressed as the relative change in a given parameter compared to the mean of the respective blank‐inoculated treatment. Treatment labels on the horizontal axis consist of the element (N or P) concerned and its input dose (zero, single, or triple). Each bar represents the mean of five replicates (±SE). Symbols above the columns indicate the significance of the contrast between the means of mycorrhizal and non‐mycorrhizal treatments for each fertilization treatment according to the *t*‐test (*0.01 ≤ *P *<* *0.05; **0.001 ≤ *P *<* *0.01; ***P <* *0.001; ns, *P *≥* *0.05). Different letters above columns indicate significant differences among fertilization treatments according to Tukey's HSD test (*P *<* *0.05). n.a. – not applicable, treatment not included in this experiment.

While mycorrhizal inoculation had no effect on TDW in the nonfertilized treatment, it significantly increased TDW in all three fertilized treatments (Fig. 3A). The R:S dry biomass ratio showed a consistent shift along the fertilization gradient: In the N0 P0 treatment group, mycorrhizal plants had significantly higher R:S ratios than did the non‐mycorrhizal plants. In the N1 P1 treatment group, the R:S ratio was similar for both inoculation treatments. In the two fertilization treatments with the triple‐N dose, higher R:S ratios were encountered in non‐mycorrhizal as compared to mycorrhizal plants (Fig. 3B). For ^13^C BGD, the same pattern was found along the fertilization gradient (Fig. 3C). The two parameters R:S ratio and ^13^C BGD were positively correlated (*P *<* *0.001; Fig. 4A). Plants’ MGR progressively increased with increasing N input (Fig. 5A) and was strongly and positively correlated (*P *<* *0.001) with the incidence of arbuscules in the roots (Fig. 4B).

#### Experiment 2

**Figure 6:**
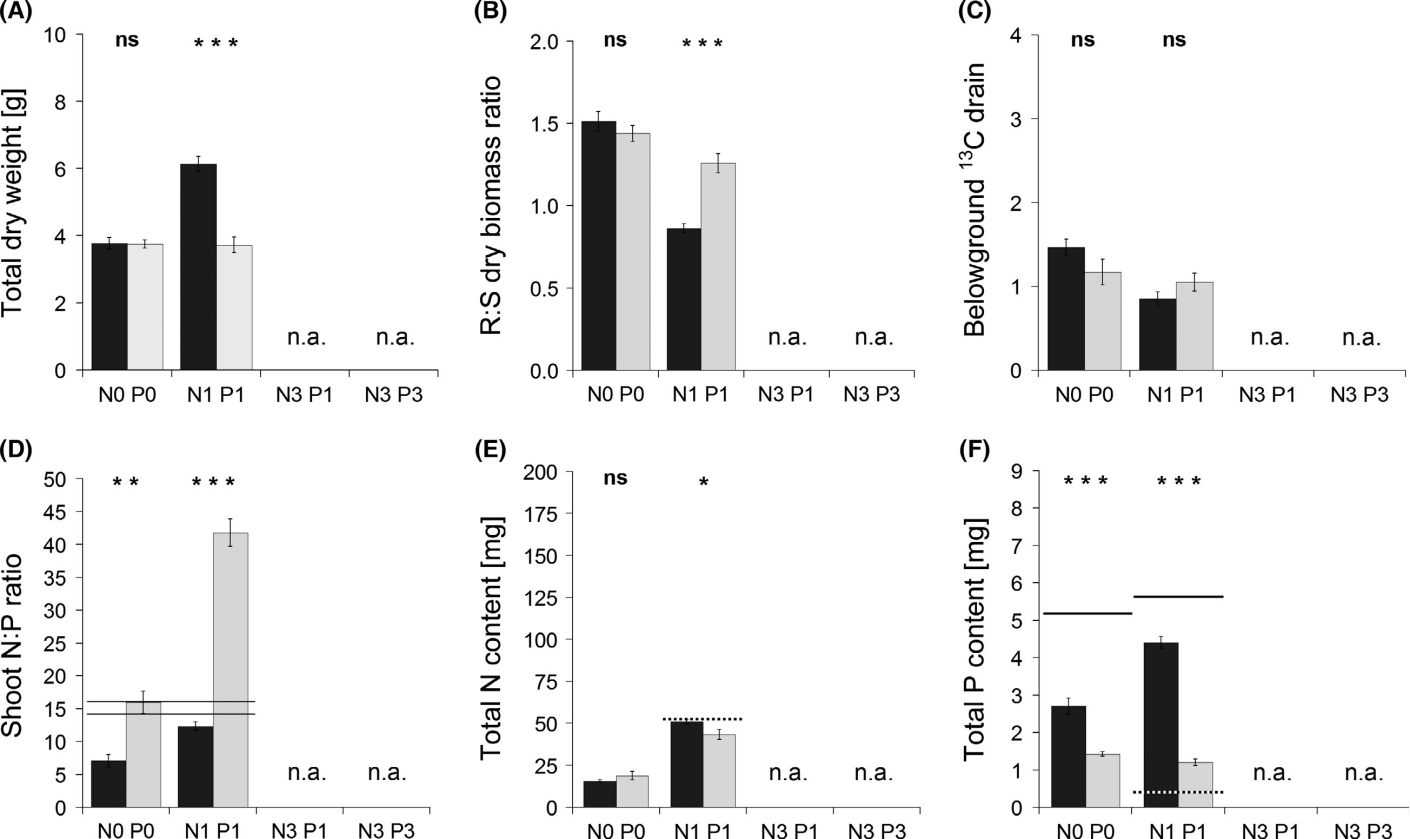
Total (root + shoot) dry biomass (A), root‐to‐shoot (R:S) dry biomass ratio (B), belowground ^13^C drain (C), shoot nitrogen‐to‐phosphorus content ratio (D), total nitrogen (N) content (E), and total phosphorus (P) content (F) of *Andropogon gerardii* plants grown in Experiment 2 under two fertilization treatments, either with *Rhizophagus irregularis* (dark columns) or with a respective blank inoculum (light columns). Treatment labels on the horizontal axis consist of the element (N or P) concerned and its input dose (zero or single). Each bar represents the mean of five replicates (±SE). Symbols above the columns indicate the significance of the contrast between the mycorrhizal and non‐mycorrhizal plants for each fertilization treatment according to the *t*‐test (*0.01 ≤ *P *<* *0.05; **0.001 ≤ *P *<* *0.01; ***P <* *0.001; ns, *P *≥* *0.05). The lower horizontal line in graph D indicates the level of shoot N:P ratio below which the plants are considered N‐limited, whereas the upper line indicates the N:P ratio above which they are considered P‐limited according to Koerselman and Meuleman (1996). Between these lines, the plants are considered to be colimited by both nutrients. The dotted horizontal lines in graphs E and F indicate the levels of N and P inputs, respectively, from the fertilizers in the fertilized treatment. The solid lines in graph F indicate the levels of available (water‐extractable) P content in the experimental pots. n.a. – not applicable, treatment not included in this experiment.

Generally, the plants in Experiment 2 showed growth responses congruent with those in Experiment 1 (Table 2; Figs. 5D and 6A–C). Inoculation with the *R*. *irregularis* isolate had no significant effect on the growth of plants in the nonfertilized treatment, but it markedly increased all growth parameters upon fertilization (Figs. 5D and 6A).

**Table 2:** Effects of the two experimental factors (Fertilization, two levels, and mycorrhizal Inoculation, two levels) and their interaction on various parameters of *Andropogon gerardii* plants grown in Experiment 2 as per the two‐way analysis of variance. *F*‐values and statistical significance levels are shown

Parameter	Factor	*F*	*P*
Total dry weight	(1) Fertilization	37.14	***
	(2) Inoculation	40.65	***
	(1) × (2)	39.35	***
Shoot dry weight	(1) Fertilization	93.87	***
	(2) Inoculation	67.39	***
	(1) × (2)	73.86	***
Root dry weight	(1) Fertilization	3.14	ns
	(2) Inoculation	12.11	**
	(1) × (2)	8.92	**
R:S dry biomass ratio	(1) Fertilization	76.45	***
	(2) Inoculation	11.68	**
	(1) × (2)	24.38	***
Belowground ^13^C drain	(1) Fertilization	10.27	**
	(2) Inoculation	0.18	ns
	(1) × (2)	4.83	*
Shoot N:P ratio	(1) Fertilization	111.09	***
	(2) Inoculation	168.58	***
	(1) × (2)	48.48	***
Total N content	(1) Fertilization	200.10	***
	(2) Inoculation	1.23	ns
	(1) × (2)	6.93	*
Shoot N concentration	(1) Fertilization	61.45	***
	(2) Inoculation	30.51	***
	(1) × (2)	7.91	*
Root N concentration	(1) Fertilization	147.74	***
	(2) Inoculation	3.13	ns
	(1) × (2)	1.85	ns
Total P content	(1) Fertilization	26.50	***
	(2) Inoculation	249.01	***
	(1) × (2)	45.90	***
Shoot P concentration	(1) Fertilization	3.68	ns
	(2) Inoculation	89.24	***
	(1) × (2)	0.24	ns
Root P concentration	(1) Fertilization	0.08	ns
	(2) Inoculation	139.32	***
	(1) × (2)	1.01	ns

*0.01 ≤ *P *<* *0.05; **0.001 ≤ *P *<* *0.01; ***P <* *0.001; ns, *P* ≥ 0.05.

### Uptake of nutrients

#### Experiment 1

Plants’ uptake of N and P was affected by both experimental factors, and the interaction between the two factors, too, was often significant. Inoculation had no significant effect on root N concentration, however (Table 1).

Nonfertilized mycorrhizal plants suffered from N limitation, whereas non‐mycorrhizal plants were slightly P‐limited. Fertilization overcame the N limitation of mycorrhizal plants, but the non‐mycorrhizal plants remained highly P‐limited even in the N3 P3 treatment (Fig. 3D).

**Figure 7:**
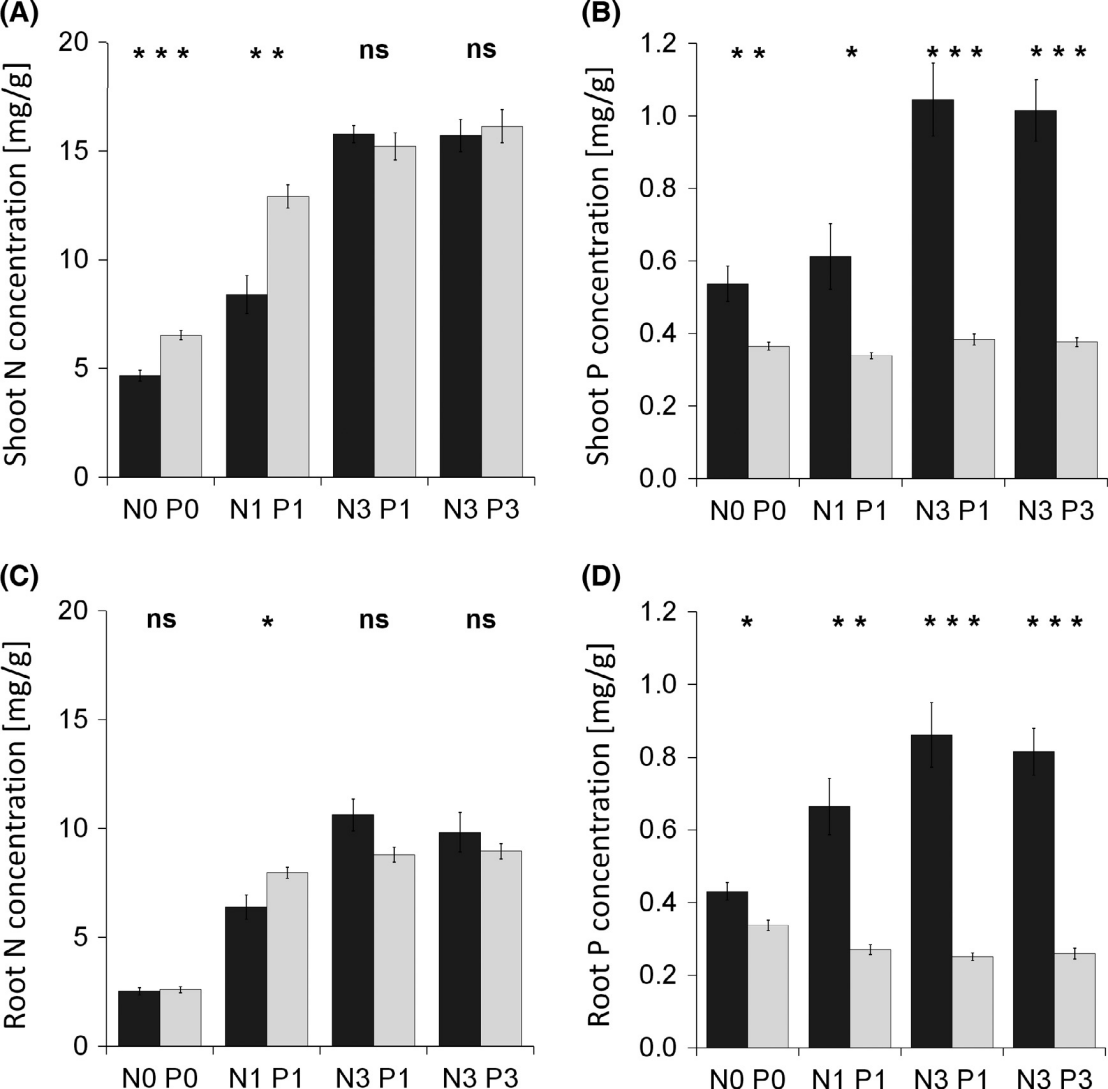
Shoot nitrogen (N) concentration (A), shoot phosphorus (P) concentration (B), root N concentration (C), and root P concentration (D) in *Andropogon gerardii* grown in Experiment 1 under four fertilization treatments, either with a native mycorrhizal community (dark columns) or with a respective blank inoculum (light columns). Treatment labels on the horizontal axis consist of the element (N or P) concerned and its input dose (zero, single, or triple). Each bar represents the mean of five replicates (±SE). Symbols above the columns indicate the significance of the contrast between the means of mycorrhizal and non‐mycorrhizal treatments for each fertilization treatment according to the *t*‐test (*0.01 ≤ *P* < 0.05; **0.001 ≤ *P* < 0.01; ***P < 0.001; ns, *P *≥* *0.05).

Mycorrhizal N‐uptake response was negative in the nonfertilized treatment, nonsignificant in the N1 P1 treatment, and strongly positive in both N3 treatments (Fig. 5B). It positively correlated (*P *<* *0.001) with the incidence of arbuscules in the roots (Fig. 4C). Nonfertilized mycorrhizal plants had significantly lower total N content than did non‐mycorrhizal plants (Fig. 3E). Moderate fertilization (N1 P1) resulted in equalization of N content in both inoculation treatments, while further increases in nutrient input resulted in a significant increase in N content of mycorrhizal plants as compared to the non‐mycorrhizal controls. In neither fertilization treatment did the plants completely use up the N inputs provided with the nutrient solution (Fig. 3E). N concentration in plant shoots was affected by the mycorrhizal inoculation only in the nonfertilized (N0 P0) and moderately fertilized (N1 P1) treatments, with significantly lower concentrations found in mycorrhizal plants (Fig. 7A). Root N concentration differed between mycorrhizal and non‐mycorrhizal plants only in the N1 P1 treatment, with mycorrhizal plants showing slightly lower concentrations than did non‐mycorrhizal plants (Fig. 7C).

Mycorrhizal P‐uptake response progressively increased with increasing N input (Fig. 5C) and also was positively correlated (*P *<* *0.001) with the incidence of arbuscules in the roots (Fig. 4D). In all fertilized treatments, mycorrhizal plants had significantly higher P content than did non‐mycorrhizal plants (Fig. 3F). Mycorrhizal plants used far more P than what was added with the respective nutrient solutions, obviously exploiting the P from substrate components (Fig. 3F). The concentrations of P in both shoots and roots were always significantly higher in mycorrhizal as compared to non‐mycorrhizal plants (Fig. 7B and D).

#### Experiment 2

Although lower uniformity in the effects of experimental factors on the measured plant nutritional parameters was found for Experiment 2 (Table 2) compared to Experiment 1, the main trends were highly congruent between the two experiments.

**Figure 8:**
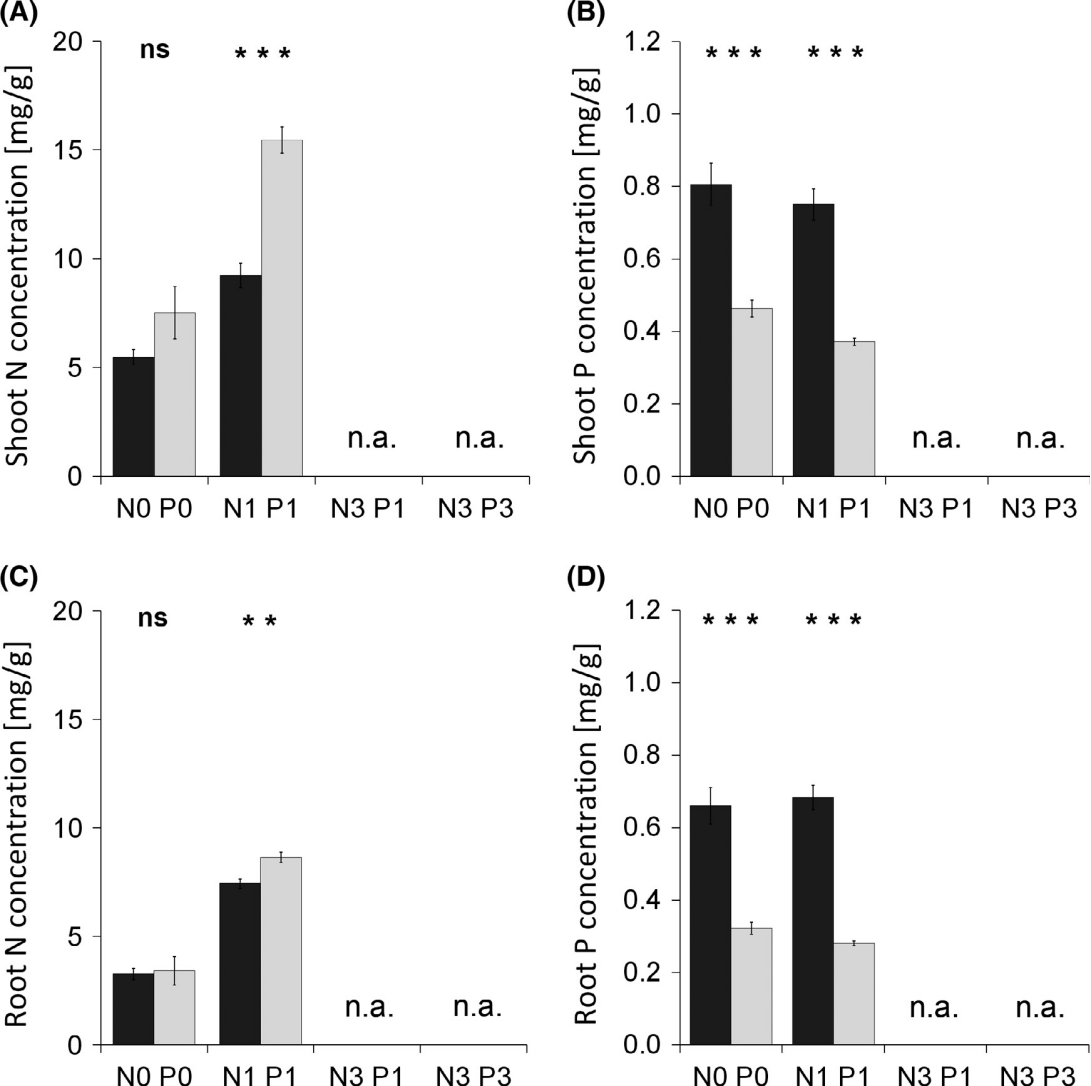
Shoot nitrogen (N) concentration (A), shoot phosphorus (P) concentration (B), root N concentration (C), and root P concentration (D) in *Andropogon gerardii* grown in Experiment 2 under two fertilization treatments and inoculated either with *Rhizophagus irregularis* (dark columns) or with a respective blank inoculum (light columns). Treatment labels on the horizontal axis consist of the element (N or P) concerned and its input dose (zero or single). Each bar represents the mean of five replicates (±SE). Symbols above the columns indicate the significance of the contrast between the means of mycorrhizal and non‐mycorrhizal treatments for each fertilization treatment according to the *t*‐test (**0.001 ≤ *P* < 0.01; ***P < 0.001; ns, *P *≥* *0.05). n.a. – not applicable, treatment not included in this experiment.

Mycorrhizal plants were generally N‐limited, whereas non‐mycorrhizal fertilized plants were strongly P‐limited (Fig. 6D). The presence of *R*. *irregularis* significantly decreased N uptake in the N0 P0 treatment (Fig. 5E), but slightly increased N uptake after the moderate fertilization (Figs. 5E and 6E). Similarly to as seen in Experiment 1, shoot and root N concentrations tended to decrease due to inoculation with *R*. *irregularis*, which difference became significant in the N1 P1 treatment (Fig. 8A and C). Also in congruence with Experiment 1, P uptake was significantly increased by inoculation with *R*. *irregularis* (Figs. 5F, 6F and 8B, D).

## Discussion

We had hypothesized that increasing the relative availability of N would increase plants’ MGR and root colonization levels, and both of these hypotheses were confirmed. In addition to the MGR, plants’ mycorrhizal N‐uptake and P‐uptake responses also increased progressively along the N fertilization gradient, congruently in both experiments. One of the most surprising results was that – identically in both experiments – non‐mycorrhizal plants were unable to translate the improved N nutrition due to N inputs into a growth increase. Most likely, this was due to the primary P limitation of their growth and their limited capacity to take up P from the substrate without mycorrhiza. Although there was plenty of light and also some available P in the cultivation substrate, non‐mycorrhizal plants remained stunted, regardless of fertilization treatment. If grown without AM fungi, plants were thus simply unable to realize any growth advantage from fertilization. That was despite the fact that there was an increase in total N uptake by the non‐mycorrhizal plants with increasing N input (Fig. 3E). Another noteworthy result was the obvious and strong competition between plants and AM fungi for N, which turned out to be the main limitation upon plants’ productivity at low N application rates. Lack of mycorrhizal growth stimulation in the nonfertilized substrate (actually, SDW of mycorrhizal plants in Experiment 1 was significantly decreased as compared to the respective non‐mycorrhizal control) revealed that under the condition of low N availability the fungi were behaving not as N suppliers, but rather as selfish hoarders. Under N limitation, the AM fungi decreased N uptake by mycorrhizal plants, which was reflected in both those plants’ lower total N content and shoot N concentration as compared to the non‐mycorrhizal controls. Only when N was added to the system in the form of mineral fertilizer did mycorrhizal functioning turn toward mutualism, consistent with the trade balance model (Johnson 2010; Johnson et al. 2015). The notion that mutualistic behavior of the AM fungi in the nonfertilized substrate degraded toward parasitism (or hoarding strategy, Kiers et al. 2011) is further supported by the fact that root colonization levels by vesicles were not affected by the fertilization treatments and remained high across all treatments (i.e., between 20% and 40% of root length colonized), whereas colonization by arbuscules (active sites of reciprocal exchanges of nutrients for carbon between AM fungi and the plants) was lower in the unfertilized than in the fertilized treatments (Fig. 2).

Similarly to the MGR, the increased availability of N resulted in progressively intensified mycorrhizal colonization of the roots. This was in accordance with our hypothesis which was based on previously reported high N demand by AM fungi (Hodge and Fitter 2010). The inputs of N applied here thus probably fitted within the range beneficial for the functioning of AM symbiosis. Whereas a low amount of soil N can clearly limit AM fungal abundance (Grman and Robinson 2013), fertilization of originally low‐fertility soil can induce shifts in the composition of AM fungal communities and eventually lead to lower levels of root colonization (Johnson 1993; Sochorová et al. 2016). The response of AM fungi to fertilization is highly context dependent, however, with the soil N:P ratio playing a particularly important role. Johnson et al. (2003, 2015) found that N addition negatively affected AM colonization of roots in soils with low N:P ratios and positively affected AM colonization in those with high N:P ratios. Johnson et al. (2015) explained this using the functional equilibrium model (Brouwer 1983) as follows: N fertilization of soils relatively rich in P causes both mineral nutrient resources to become amply available and not limiting plant growth. Thereafter, plants can invest more resources (such as C) into shoots and less into roots and mycorrhizas. In N‐rich and P‐deficient soils, the additional input of N further increases the imbalance between resources, P becomes even more limiting, and plants are forced to invest more into the roots and AM fungi. The N application gradient applied in the study of Johnson et al. (2015), equivalent to 25, 50, and 75 kg N per hectare, at least partly overlaps with the N application rates in our study (55 and 160 kg N per hectare). Nevertheless, we observed a directly opposite, stimulatory effect of N application on AM colonization. This most likely is due to the fact that while the soil used by Johnson et al. (2015) provided ample amounts of available P, plants in our study were more P‐limited. The results thus fit well into the trade balance model proposed previously (Johnson et al. 2015).

Further, we had expected that increasing P availability would decrease both the MGR and mycorrhizal colonization of roots. These hypotheses could not be confirmed based on our experimental data. One possible explanation is that the levels of P applied in this experiment (0.43 and 1.29 mg of total P input per pot) were too low as to negatively affect mycorrhizal development and/or plant response to mycorrhiza development. That is to say that increase in P supply in severely P‐deficient soils may even increase mycorrhizal colonization and/or benefits to plants (Bolan et al. 1984; Propster and Johnson 2015; Teste et al. 2016) and only abundant P inputs would negatively affect the development of AM symbiosis and/or its benefits to the host (Hetrick et al. 1986; Treseder and Allen 2002). Therefore, much greater P application rates might be needed to reduce root colonization levels and/or mycorrhizal benefits to the plant in our experimental system.

Finally, we hypothesized, based on the trade balance model, that plants with the highest MGR would invest more C into their AM fungal symbionts, thus reciprocating for the mycorrhizal benefits provided (Johnson 2010; Kiers et al. 2011). Surprisingly, belowground C drain did not correlate with MGR, but rather with R:S plant biomass partitioning. If highly N‐fertilized and mycorrhizal plants “outsourced” their mineral nutrition to AM fungi (as indicated by the highest N‐ and P‐uptake mycorrhizal responses), they could, consequently, invest less C into the root system and favor the shoots instead (their R:S ratio was < 1), thereby maximizing their photosynthesizing parts. On the other hand, the symbiosis never comes for free and we would expect a significant amount of C to be passed on to AM fungi. Therefore, the low amounts of C allocated to belowground are quite surprising and could be a sign that some important component was missing in our C budget. Most likely, this included C from belowground respiration, which is quite variable depending on roots’ mycorrhizal status and could make up about the same amount of recently fixed C as did the root biomass itself (Grimoldi et al. 2006; Lendenmann et al. 2011). On the other hand, this could also indicate that the symbiosis has rather low absolute C costs. It seems that the C flux to AM fungi in our experiment was well below the 20% reported for young cucumber plants (Jakobsen and Rosendahl 1990), and possibly closer to the 8% of the net primary production reported for another grass species (Grimoldi et al. 2006). In addition, photosynthesis in mycorrhizal plants could also be upregulated, effectively offsetting the C costs incurred (Kaschuk et al. 2009, 2010). In any case, the exact numbers need to be generated while taking into account such additional compartments of the experimental system as the C respired from the below‐ and aboveground parts. In addition and as noted previously (Wright et al. 1998; Lendenmann et al. 2011), differences in size and/or nutritional status of the differently treated plants can effectively hide potential shifts in the C budget among experimental treatments. Solving this conundrum will be anything but easy.

An important aspect of our study was to corroborate the congruency of the results between the two experiments inoculated with the complex soil inoculum containing several different AM fungal species and many other microbes on the one hand and a single AM fungal isolate on the other. This indicates that no particularly devastating pathogens were either present in the field soil or able to develop under the experimental conditions applied here. Mycorrhizal symbiosis was unambiguously the primary determinant of the soil–plant feedback in our model system.

We can conclude that our findings clearly demonstrate the importance of sufficient N supply for mutualistic functioning of AM symbiosis. Although AM symbiosis promotes N uptake by plants, this happens only if sufficient N is present in the substrate. Under conditions of N deficiency, the net effect of AM fungi could easily spiral down into negative values, with the AM fungi effectively decreasing plant uptake of N and erasing mycorrhizal growth benefits in such a highly mycorrhiza‐responsive plant as *A. gerardii*.

## Conflict of Interest

None declared.
